# Media framing of emergency departments: a call to action for nurses and other health care providers

**DOI:** 10.1186/s12912-021-00606-2

**Published:** 2021-07-04

**Authors:** Kimberley Thomas, Annette J. Browne, Sunny Jiao, Caryn Dooner, Patrice Wright, Allie Slemon, Jennifer Diederich, C. Nadine Wathen, Vicky Bungay, Erin Wilson, Colleen Varcoe

**Affiliations:** 1 Faculty of Medicine, The University of British Columbia, Vancouver, BC Canada; 2 School of Nursing, The University of British Columbia, Vancouver, BC Canada; 3 The Faculty of Graduate and Postdoctoral Studies (Public Health/Nursing), The University of British Columbia, Vancouver, BC Canada; 4 Arthur Labatt Family School of Nursing, Western University, London, Ontario Canada; 5 Capacity Research Unit, School of Nursing, The University of British Columbia, Vancouver, BC Canada; 6 School of Nursing, University of Northern British Columbia, Prince George, BC Canada

**Keywords:** Emergency Departments, Nurses, Nursing, Media, Health Equity, Social Justice, Stigma

## Abstract

**Background:**

As part of a larger study focused on interventions to enhance the capacity of nurses and other health care workers to provide equity-oriented care in emergency departments (EDs), we conducted an analysis of news media related to three EDs. The purpose of the analysis was to examine how media writers frame issues pertaining to nursing, as well as the health and social inequities that drive emergency department contexts, while considering what implications these portrayals hold for nursing practice.

**Methods:**

We conducted a search of media articles specific to three EDs in Canada, published between January 1, 2018 and May 1, 2019. Media items (*N* = 368) were coded by *story* and *theme* attributes. A thematic analysis was completed to understand how writers in public media present issues pertaining to nursing practice within the ED context.

**Results:**

Two overarching themes were found. First, in ED-related media that portrays health care needs of people experiencing health and social inequities, messaging frequently perpetuates stigmatizing discourses. Second, media writers portray pressures experienced by nurses working in the ED in a way that evades structural determinants of quality of care. Underlying both themes is an absence of perspectives and authorship from practicing nurses themselves.

**Conclusions:**

We recommend that frontline nurses be prioritized as experts in public media communications. Nurses must be supported to gain critical media skills to contribute to media, to destigmatize the health care needs of people experiencing inequity who attend their practice, and to shed light on the structural causes of pressures experienced by nurses working within emergency department settings.

## Background

In Canada, despite a nationally funded health care system, health and social inequities are increasing [1]. Emergency departments (EDs) play a critical role in providing care to people who experience significant health and social inequities, frequently extending their roles beyond the essential acute care services they are designed to provide. For this reason, many health care providers in Canada frame emergency departments as “a cornerstone of Canadian health care, providing universal access to care for all patient presentations, including underserved and/or disadvantaged populations, at all times” [2]. In addition to being first-contact points of care for emergency services, EDs in Canada tend to be accessed by people who are seeking help for unmet primary care needs, particularly when community-based primary care services are not available, are perceived as inaccessible, or when people have negative experiences obtaining care in the primary care sector [3, 4]. The erosion of the social safety net in the pre-COVID-19 era has had particularly deleterious impacts for people experiencing multiple intersecting inequities including, for example, Indigenous peoples, people living with stigmatizing and complex chronic conditions, women experiencing intimate partner violence, people for whom safe affordable housing is not available, and people with lived experience of substance use stigma [1, 4–7]. These contextual factors contribute to and intersect with system-wide pressures related to overcrowding, long wait times, and overcapacity in EDs in Canada [8, 9].

How media represents health care and health care providers influences people’s perceptions and use of the ED, yet such representation is often not well informed by those most directly involved. The landmark Woodhull study conducted in the late 1990's examined media articles related to health and found that, for example, only 4 % referenced nurses [10] despite nurses comprising the majority of health care workers. In a replication study twenty years later, nursing was mentioned in 13 % of articles, though nurses were the source of only 2 % of all quotations, largely discussing the nursing profession itself rather than issues related to patient care or the health care system [11]. These findings illustrate a critical gap in nursing’s representation in media. Yet, members of the nursing profession must examine not only *whether* nurses are represented, but *how* nurses, and other staff, can meaningfully engage with media to shape and challenge hegemonic discourses. Given nurses’ position as the largest group of health care providers in the ED and their role in providing direct care, there remains significant unrealized potential for nurses, and other clinical staff, to directly contribute their voices to media and shape public conversations about EDs and the broader health care system. The framing of health and social inequities, and the specific content, perspectives, and language used can move media along a spectrum of acting as an agent for change or as a reinforcement of prevailing stigma and the status quo [12].

This project fits within a larger organizational intervention, EQUIP Emergency, a study designed to enhance capacity for equity-oriented care in EDs in three geographically and demographically different cities in the province of British Columbia (BC), Canada. A detailed overview of EQUIP Emergency is provided in a previous publication [13]. One of the aims of EQUIP Emergency was to orient the ED clinical leaders and staff to the broader sociopolitical and community contexts of the local populations served. To achieve that aim, the EQUIP Emergency research team developed socio-contextual profiles for each of the three EDs. As part of building these context profiles, our team conducted a targeted analysis of media articles written about the three participating EDs to better understand the range of messaging in public media, and to examine those through a health equity lens. The purpose of the present analysis is to examine how writers in public media frame issues concerned with health and social inequities in relation to EDs and the hospitals and wider communities in which the EDs are positioned, as well as to consider implications of this analysis for nurses and other health care providers, leaders, and staff. Our analysis was guided by the following research question: How are issues pertaining to health and social inequities in relation to EDs framed in media, and what are the implications of this for nurses and others working in health care?

This examination offers an opportunity to understand external factors shaping emergency care nursing practice and raise awareness about the potential for clinical staff to contribute to public narratives in ways that counteract stigmatizing discourses that affect patient health outcomes and quality of care. During our analysis process, and through a critical social justice lens, we observed that the voices and perspectives of staff, especially nurses, were often absent in mainstream media items, consistent with previous analyses [10, 11]. We believe it is important to increase the visibility, in ED- and health-related media articles, of those providing the majority of direct patient care, and our paper aims to empower nurses as the largest ED workforce, as well as other clinical staff, to help shape public conversations towards changing perceptions.

Although our original analysis occurred before the onset of the COVID-19 pandemic, considering the current evolving circumstances, we also use this paper as an opportunity to examine how health and social inequities exacerbated by the pandemic are being framed in media. This paper will begin by outlining the theoretical perspectives, methods, and community contexts of the EQUIP ED study sites. We discuss the two overarching themes identified through our analysis and end with implications for nursing practice, and the relevance of media framings to the ongoing COVID-19 pandemic. The article will conclude with recommendations for action at practice and policy levels.

## Methods

### Theoretical perspectives

This media analysis is informed by a critical social justice lens, which supports the examination of the power dynamics and imbalances that underlie systemic or structural inequities such as inequities in health and in health care [14]. This lens takes us “beyond the distributive justice paradigm […] and beyond the rhetoric of […] equal treatment for all” [15] and allows us to discover “ways in which […] forms of oppression support unjust arrangements of social goods” [14]. Hence, instead of thinking about achieving equality, where the goal is to treat everyone the same, we shift to a focus on achieving equity. Achieving equity requires thinking about how to improve access and service provision for people who have the greatest health needs and the most difficulty accessing care.

The analysis we discuss in this paper is informed by current conceptualizations of health equity as a social justice goal focused on pursuing the highest possible standard of health and health care for all people, paying special attention to those in the context of greater risk of poor health, and taking into account broad social, political, and economic influences and access to care [16, 17]. We further define equity-oriented care as an approach that aims to reduce the negative health effects of: structural inequities and structural violence; the multiple and intersective forms of racism, discrimination and stigma; and the frequent mismatches between usual approaches to care and the needs of people most impacted by health and social inequities [3]. In applied health contexts such as nursing, critical perspectives about social justice and the ideas underpinning equity-oriented health care can provide a useful lens through which to consider nursing’s role and responsibility in shaping public messages and understandings regarding health equity issues.

### Study context

This analysis examined media related to three EDs in different cities within the province of British Columbia, Canada: (1) St. Paul’s Hospital (SPH) in Vancouver – the “Urban” ED; (2) Surrey Memorial Hospital (SMH) in Surrey – the “Suburban” ED; and (3) University Hospital of Northern BC (UHNBC) in Prince George – the “Northern Regional” ED. Each hospital is uniquely geographically located and serves a diverse community. Table 1 provides an overview of some of the key features of the community contexts in which each hospital is located. As will be discussed in the analysis, these contextual features provide important insights into why certain themes are featured in media items related to specific sites at particular points in time, as well as to why health equity has become an important goal in the provision of care at each site.

**Table 1:** Community & Emergency Department Context of Three Sites of Media Analysis

Study Site	Community & Hospital Context	ED-Specific Context
“Urban ED” St. Paul’s Hospital (SPH) Vancouver Coastal Health Authority	▪ Located in the west end of a large metropolis in Western Canada on unceded traditional lands of the xʷməθkʷəy̓əm (Musqueam), Skwxwú7mesh (Squamish), and Səl̓ílwətaʔ/Selilwitulh (Tsleil-Waututh) Nations. ▪ Primary hospital for an urban area and adjacent neighbourhoods, including the inner-city community commonly referred to as the Downtown Eastside^a^. ▪ Extensive expertise providing care to patients who experience challenges related to substance use, often related to the opioid epidemic^b^.	▪ The SPH ED serves over 80,000 patients annually and up to 300 patients per day [18]. ▪ The SPH ED plays a critical role in responding to the opioid crisis, in terms of treating people who have overdosed, and also houses an overdose prevention site [19].
“Suburban ED” Surrey Memorial Hospital (SMH) Fraser Health Authority	▪ Located on the lands of the Semiahmoo, Katzie, Kwantlen, Tsawwassen, QayQayt and Kwikwetlem First Nations in a suburban setting in the city of Surrey, the fastest growing city in BC. ▪ About one in four Surrey residents live in poverty. ▪ SMH serves a relatively high proportion of new immigrants and refugees.	▪ The largest ED in Western Canada. ▪ Over 165,000 people visited the SMH ED in 2018, making it the busiest ED in the province of British Columbia [20].
“Northern Regional ED” University Hospital of Northern British Columbia (UHNBC) Northern Health Authority	▪ A central-interior regional hub located on the traditional territory of the Lheidli T’enneh First Nation. ▪ Level III trauma centre and the largest hospital in the region, providing services to people dispersed over an area of approximatively 600,000 km^2^. ▪ UHNBC serves a relatively high proportion of Indigenous^c^ peoples. ▪ There are 54 First Nations communities within Northern Health [21].	▪ Advanced referral ED for over 300,000 regional residents [22].

^a^The Downtown Eastside (DTES) is a community built upon tremendous support and co-existence amongst people who include, for example, people who live in extreme poverty, people who lack access to safe and affordable housing, and women who are disproportionately affected by intimate partner violence

^b^The opioid epidemic is a major public health crisis in British Columbia due to unintentional drug overdoses. Opioid-related overdoses and deaths have increased since 2011, rose dramatically in 2015, and a public health emergency was declared in 2016; this public health emergency remains in place [23]

^c^Consistent with accepted terminology used in landmark international reports, the term ‘Indigenous peoples’ is used to refer to the diversity of populations throughout the world. In Canada, over 1.7 million people of the total population of ~34.5 million (4.9%) identify as Indigenous [7], including First Nations, Métis, and Inuit people. In British Columbia, the Northern Health Authority (where the "Northern Regional ED" is situated), has the highest proportion of Indigenous peoples amongst all health authorities in British Columbia. Approximately 20.1% of the population served by the Northern Health Authority self-identify as Indigenous [24]

### Thematic analysis of media articles

We conducted a systematic search of media items, published in the English language, about the three hospitals and their respective EDs between January 1, 2018 and May 1, 2019. In consultation with a health sciences librarian, a comprehensive search strategy was developed to capture mainstream and local media items for each site. Search terms included the names (including variants and abbreviations) of each hospital and the respective health authority. Databases searched included Canadian Newsstream, which indexes publications from national and local news outlets, and five local Indigenous news outlets that are not indexed in Canadian Newsstream. Articles that included the hospitals’ names in birth announcements or obituaries were excluded. Our search identified 368 total articles for examination: 248 articles related to SPH (the Urban ED), 36 articles to SMH (the Suburban ED), and 84 articles to the UHNBC (The Northern Regional ED).

A thematic analysis was conducted to examine how media frames issues of health and social inequities within EDs and the broader context of health care [25]. Media items were first coded by *story* and *theme*. The *story* attribute recognizes that a news story is often covered by multiple news or media outlets. The *theme* for each media item refers to the central concept or issue discussed within the article, and this was identified by reading the article through the lens of critical social justice and equity-oriented care. For example, a prominent newspaper (*The Vancouver Sun*) featured a story about a new program at the Urban ED providing people who have experienced an overdose with a three-day Suboxone prescription. This story was publicized 20 times across different media outlets. To capture the context and content of the story, this article was coded with the broader theme of “Responses to the Opioid Crisis”. The coding process was an iterative process undertaken by first analyzing written media articles pertaining to each ED separately to ground analysis in context. Themes were then reviewed by the entire research team across the three contexts and revised to capture broader themes of media representation of issues pertaining to health and social inequities.

**Fig. 1:**
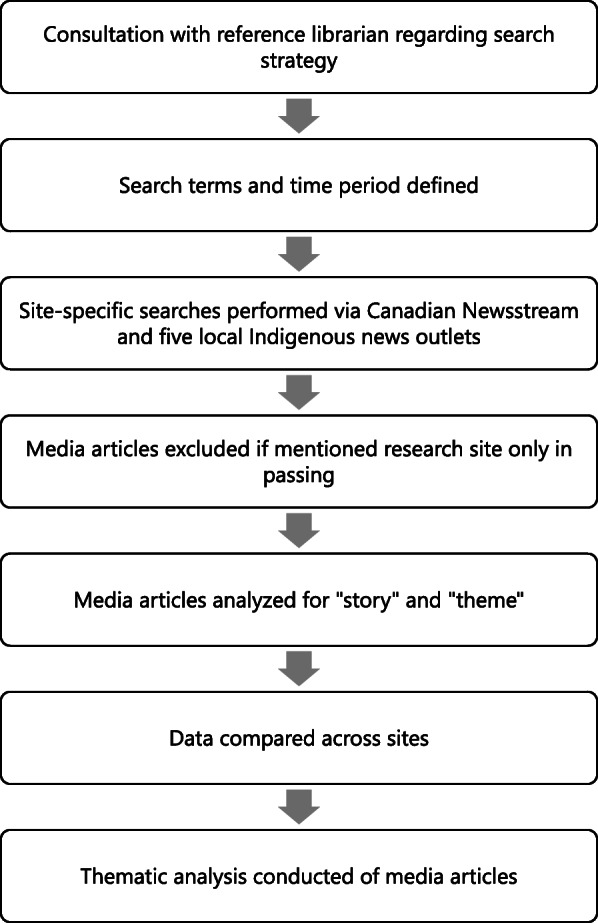
Flow Diagram for Media Search Strategy

Following the thematic coding process, specific themes and the media items within that theme were analyzed using a critical social justice lens. While not all media items identified in our search related to inequities in the ED context, this analysis focuses on media items with equity implications such as “Cultural Safety”, “Drug Use and Substance Use”, and “ED Wait Times”. Equity implications were identified by determining whether stories were related to health equity or inequity, such as unjust or potentially remedial differences in health or access to care [26]. Full text articles were analyzed to gain insight into how media frames issues of health and social inequities in the ED, contextual factors shaping inequities, approaches to equity-oriented care within the ED setting and broader health systems, and nursing and other clinical staff roles in enacting equity-oriented care. An analysis of these media articles informs our discussion of media discourses and their potential influence on shaping the public’s broader understandings of health equity issues. As we discuss below, this has important implications for nurses and other health care staff in helping to re-shape public conversations. A flow diagram providing an explanation of the media search strategy and how we achieved our final data set is shown in Fig. 1.

## Results

Our analysis identified two overarching themes related to how existing health and social inequities are framed in media specific to our ED sites. First, in ED-related media that portrays health care needs of people experiencing health and social inequities, messaging frequently perpetuated stigmatizing discourses. Second, ED-related media items portrayed pressures experienced by the ED in a decontextualized way, without attention to root, structural causes. Underlying both themes is a clear absence of representation, voice, and authorship from nurses and other direct service providers working in EDs.

### ED-related media perpetuates stigmatizing discourses

The language, perspectives, terminology, and images featured in media items have the potential to both perpetuate and counteract stigmatizing discourses. For example, a prominent focus of media items before (and during) the COVID-19 pandemic was on the issue of ED overcapacity, in which the numbers of people seeking care exceeds ED beds and staffing capacity. In our analysis, articles that focused on overcapacity often included messaging that used language to attempt to divert people away from receiving care in the ED. In the following example, the writer relays messages sent by the regional health authority about overcapacity in the Northern Regional ED:

“It’s only been two months since Northern Health sounded the alarm over University Hospital, but they’re doing it again. It says the entire hospital is full, not just the Emergency Room. And there’s not one single thing to pin it on, such as influenza. And Northern Health is asking people to steer clear if they can” [27].

Relatedly, our analysis also revealed that media reinforced ideas about “appropriate” use of the ED, for example, during our search of articles related to the Northern Regional site, we found media headlines such as “Too Many Patients at UHNBC” [28] and “Unnecessary hospital trips clogging up Fort St. John ER” [29]. Although overcapacity and overcrowding at these EDs are factual realities [30], the broader contexts of people’s lives that influence their use of EDs (e.g., inadequate and unsafe housing) tends to be overlooked [31–33]. Headlines such as these imply that patients have the choice to visit the ED or not, and this is not necessarily true for everyone. Such diverting discourses minimize or ignore the fact that even in countries with well-resourced health systems, people may not be able to access care other than at the ED [34].

Commonly proposed solutions to addressing the issue of ED overcapacity (such as diverting people away from using the ED) [35] frame demand for ED services as greater than what staffing levels or system resources may permit, and this only addresses the problem at a surface level. These types of solutions do little to acknowledge the root causes of why EDs are at overcapacity, and how these root causes are inseparable from issues of inequity and social justice [36]. Diversion messaging disadvantages^1^ those who seek care in EDs for complex health and social needs due to a lack of responsive services and resources in the community [37]. Messaging about the “appropriate use” of EDs also disadvantages people who have had previous experiences of discrimination in health care settings, reinforcing an already existing reluctance to seek care [38] – which in turn exacerbates morbidity, mortality and worsening health outcomes. Although in the current COVID-19 context, messaging is beginning to emerge around the dangers of delays in seeking care at EDs, people are typically encouraged to seek help at EDs for issues deemed ‘legitimate’ and therefore warranting a visit to an ED [39]. In addition, the existing messaging only notes non-attendance and delays in care due to fears of COVID-19, leaving reasons such as stigma unacknowledged and unexplored [40].

The media items we reviewed also perpetuated stigmatizing discourse by using specific language to refer to people who experience health and social inequities. We observed a distinct difference between the language used by media writers and the language used by health authorities and their representatives who were quoted by media writers. For example, in an article discussing the Suburban ED, the media writer used the word “homeless” throughout the article to refer to people who lack access to housing [41]. In contrast, quotes of a local Health Authority representative in the same article use terms such as “social housing” or “transitional housing” [41] pointing to the structural influences rather than only the people being affected by those influences. Collins [41] cites a published study, stating “52 % of 602 homeless people” use this ED “regularly,” then proceeds to start a quote with the word “they.” This type of othering language that is tied to stigmatization was also used when writers referred to people who use substances. Articles tended to use the term “drug users”, for example: “How many *drug users* who OD’d have brain damage? Doctors say Canada needs data” [42], and “Fighting a scourge in Vancouver: Determined to stop overdoses in Downtown Eastside, advocate for *drug users* broke the law. But what she did worked” [43]. Some people use the reference of “alone users”, such as “Vancouver co-op develops tech to help prevent ODs, especially for *alone users*” [44].

Othering, stereotypes, and stigmatization are entrenched and internalized through language used in mainstream media [45]. Health care institutions, organizations, and providers, including nurses working within those institutions, have the potential to influence and counteract stigmatizing discourses though challenging messaging perpetuated through public media. If not addressed, these types of damaging and dehumanizing portrayals compound with other issues (e.g., lack of transportation, language, and cost barriers) as reasons that people avoid accessing care; this can also result in policy solutions that exacerbate prevailing stigma, and further perpetuate inequities such as increased criminalization of people who use substances [46, 47]. These examples help to illuminate how language and terminology are used in ways that can be dehumanizing and othering (i.e., the process of viewing a group of people as intrinsically lesser than others). The label “substance users”, for example, is not a neutral description. Instead, person-centered language such as “people who use substances” is preferable when describing activities that are stigmatizing [4, 48]. Advocates, including groups of people who self-identify as using drugs, have consistently called for media to adopt “person-first language”, which aims to reduce stigma by centering the person rather than a behaviour or condition, and to avoid dehumanizing labels [49]. In 2017, a group of Canadian advocates and researchers sent an open letter to the Canadian Broadcasting Corporation (CBC), one of the country’s leading media outlets, with a call to use person-first language exclusively when discussing people who use drugs [50]. While the CBC adopted new language guidelines in April 2020, media items captured in this analysis frequently use stigmatizing terms, and other media outlets continue to perpetuate stigmatizing discourses through using problematic (and often pathologizing) language when speaking about people who experience health and social inequities [51–54].

Despite widespread use of stigmatizing language, some media writers countered messages of stigmatization. This alternative approach was exemplified by, for instance, an article penned by Hon. Judy Darcy, the province’s Minister of Mental Health and Addictions [55], which discussed people affected by the opioid crisis in Vancouver. Phrases in this article such as “Are we willing to stop treating mental illness and addiction as a character failure?” and “Close Collaboration with First Nations and Indigenous communities is especially vital” [55] serve to dispel misconceptions and break the cycle of stigmatization. These subtle changes in language and messaging exemplify the kind of counteracting discourses that nurses and other direct service staff can use not only in clinical practice to promote equity within health care, but also in contributing to public media. As media draws on direct quotations from health care providers including nurses, the above example illustrates how using respectful person-first language can disrupt stigmatizing discourses currently operationalized within ED-related media.

### Media portrayals of EDs ignore structural context

The second theme we focus on pertains to how media portrays pressures experienced by EDs and by the health care system at large. This theme developed through our observation that when media articles discuss EDs, it is often in relation to how busy they are. For example, one article published in a local paper, *The Peach Arch News* [56], states that the Suburban ED is the “busiest emergency room in the province” and emphasizes the need to “take pressure off the hospital emergency wards.” Another article about the Suburban ED conveyed that there was a “mess” in the ED and focused on a patient’s report of waiting in a hospital hallway for days [57]. The media also tends to use extreme words such as “crisis” in the context of EDs [58]. In these examples, structural contributors to ED environments operating at overcapacity and under extreme pressure are not mentioned. Some of these omitted root contributors include the inadequacy of health and social services in the community setting for people who experience inequities, and stigmatization in health care settings. In turn, these contributing factors are associated with delays in obtaining care as well as reliance on and repeat use of EDs [4, 59, 60].

We also identified that in stories about patients receiving poor care, blame for inadequate care was consistently placed on health care providers, most notably nurses, with little to no acknowledgement of systemic factors that greatly affect care quality or links to the aforementioned “busyness”. One systemic factor that is not sufficiently taken up by media is structural violence. Structural violence refers to the “social structures – economic, political, legal, religious, and cultural – that stop individuals, groups, and societies from reaching their full potential” [61]. The concept of structural violence brings attention to the insidious and overt ways in which systems, policies, and social dynamics such as racism and discrimination can function as sources of violence and create significant harms for people. In contrast, media discourses that centre blame and fault on individuals often fail to acknowledge the impact of structural violence. For example, an article published about the Urban ED and written from the perspective of a previous patient alludes to how nurses are to blame for a negative patient-staff encounter they witnessed in the ED:

“I recognized elements of my recent psychiatric patient experience at St. Paul’s Hospital. I got to see patients grabbed by security – on nurse’s orders – and tossed into the unit’s locked cell. Then I could hear the screams of my fellow patients as a belligerent nurse stood at the door demanding better ‘behaviour’” [62].

Since the onset of the COVID-19 pandemic, the media portrayal of “frontline staff” including nurses has notably shifted from discourses about individual fault, such as the one above, to predominantly portraying nurses as ‘heroes.’ As Einboden [63] explains, “popular news media and even medical leadership are producing and cementing an ideology that constitutes health care workers as the heroes in a war between COVID-19 and humankind”. Despite this shift, nurses are challenging and criticizing the “hero stereotype” because it portrays nurses as expendable and self-sacrificing [64, 65]. This is yet another example of how media outlets can neglect to note the greater structural context, whereby the focus should not be on how nurses are self-sacrificing, but rather on how the system has a responsibility to ensure that mechanisms and resources are in place to support staff in helping those who are already experiencing inequities and are most affected by the pandemic. Portraying nurses as self-sacrificing heroes is problematic in the same way that focusing on individual-level fault is problematic: both neglect systemic factors as root causes of poor quality of care.

Articles rarely include direct quotes or authorship from health care providers, especially nurses who are working in EDs. This is one factor that may contribute to perpetuating discourses that place fault on individual providers, as well as narratives that portray nurses as expendable and self-sacrificing. An absence of nursing representation in media can perpetuate issues of distrust and misunderstanding of the nursing profession, which in turn, can affect the likelihood of patients accessing timely care, particularly people who are already facing intersecting forms of vulnerability [66]. In the following example from an article about the Suburban ED, all direct quotes solely come from upper-level spokespeople and the premier, rather than those involved with direct care:

“Fallout continues over allegations that homeless patients were discharged from Surrey Memorial Hospital and shuttled by taxi to Chilliwack shelters. Premier John Horgan described the allegations from Chilliwack Mayor Ken Popove sent to Fraser Health as ‘startling’ during his weekly media availability Thursday in the legislature in Victoria” [67].

Greater representation and inclusion of the perspectives of nurses and other health care providers might be an effective way of shaping public perceptions, as well as bringing attention to health care system issues that result from systemic failures, rather than individual ones. We suggest that health care providers be attentive to and think critically about how the notion of efficiency is being applied to the health care system and propagated through the media, such as through discourses of overcapacity. Direct care staff should have a part in exposing how blaming discourses can serve to alleviate responsibility from the system and reallocate this responsibility onto direct care providers. Staff can also advocate for systemic structures, such as policies, to be acknowledged and addressed as causing and perpetuating harms. Lastly, they may be cautious of discourses that commend their capabilities while insidiously and implicitly denying the need for support and perpetuating the shifting of risk. While media messaging that portrays nurses and other clinicians as ‘heroes’ may generate widespread applause and public approval, this type of messaging negates health care providers’ need for structural supports that help to promote their safety.

## Discussion

### How nurses can influence media representation

Our analysis, both before and after the onset of COVID-19, surfaced articles that demonstrated how the media continues to perpetuate stigmatizing discourses, fails to acknowledge the root, structural causes of health and social inequities, and presents decontextualized portrayals of events that offer no mention of the underlying issues that produce them. Nurses hold a uniquely valuable role in counteracting stigmatizing discourses and in shaping equitable care provision [68]. As news and media can serve as crucial mediums through which advocacy occurs, nurses have the potential to participate and contribute to this process. Even more, nurses can contribute to and challenge media as a platform to bring attention to inequities and advocate for solutions to reduce and eliminate them. Increasingly, we are seeing that the support systems upon which people who experience health and social inequities rely are closing or are unavailable [69]. Nurses have a responsibility to bring attention to this important issue and to spread the word about potential impacts. In other words, nurses have an important role to play in media advocacy. In the case of COVID-19, nurses have the potential to advocate and disseminate messaging about the many health and social inequities faced by patients they care for as well as how COVID-19 is further exacerbating and perpetuating these inequities. Today, we are seeing nurses doing just this, through shedding light on inequities that have intensified and advocating for solutions from providing adequate and safe housing for people experiencing homelessness to increasing access to testing [37]. There still exists significant gaps between the permitted freedoms of expression held by nurses working in academic settings and those who are employed as direct care staff under a health authority. Academic researchers can quite freely criticize the health care system, in the public interest, through social media and news outlets, whereas direct care staff often face restrictions and backlash when doing so, even so far as being subject to disciplinary action by the court for professional misconduct [70]. A strengthening of partnerships between researchers and practicing nurses is a way of increasing the visibility of nurses who may not have the time or media literacy to craft public messages.

Despite the importance and need for nurses to highlight situations of magnified inequity in public media, nurses have faced barriers from their employers and regulatory bodies that prohibit or restrict their involvement with public media [71]. Nurses, especially those employed by the health authorities, may be subject to ‘gag orders’ and warnings that prevent them from speaking publicly and freely. Their freedom of expression may be further hindered by professional bodies, which means that they must oblige to the requirements of whichever entity has the least restrictive policy when it comes to speaking publicly about issues and influencing change. For instance, in the Canadian context, the head of one of the provincial nurses’ unions stated:

“My message to all nurses is this: please focus on your patients and providing the best quality patient care you can and let the provincial health officer focus on the message to the public. […] I’m asking every one of our members at this time to refrain from posting public messages of this nature, even if they are intended to reinforce the message of the public health officer” [72].

We recommend that there be no mandating that nurses refrain from contributing to public messaging and no abridgement of freedom of expression. Rather, there should be clear guidelines for nurses to follow that call for ethical, accurate, and non-fear-inciting messaging. For nurses who wish to relay true accounts of their experiences of care provision, there should be guidance about how to do so. While there are existing guidelines about how nurses should use personal social media accounts while upholding principles such as confidentiality, professional integrity, and public trust [73], there is limited guidance about how to share information through public media news outlets. Nurses may also be hesitant to advocate via public media for fear of reprimand from upper management or because of prohibitive workplace policies and procedures. Nurses may face bureaucratic barriers such as having to speak through professional communication channels, such as Communication Officers, that hold control over what topics are shared with media and that create a barrier between clinical practice and the outside world. A more encouraging approach might be that which is done often within post-secondary institutions, where journalists locate an expert to help bring context and insights to media reports. Health care administrators, communications departments, and public relations departments should support nurses and others in direct care roles to speak openly and honestly from their perspectives.

### Implications of findings within the current COVID-19 context

Although our analysis was conducted prior to the onset of the COVID-19 pandemic, in light of recent events, addressing issues of gross de-contextualization and insufficient acknowledgement of the underlying power structures that contribute to health and social inequities has become even more pressing. The Pan American Health Organization and World Health Organization [74], recently emphasized how inequities are exacerbated in the context of COVID-19 through deepening patterns of stigma and discrimination, including through risks to women experiencing intimate partner violence, and significant threats to older adults living in long-term care facilities, people with disabilities, people who experience homelessness, refugees, migrants (especially migrant agricultural workers), and people who are incarcerated. For example, due to complex and often intersecting social determinants of health, and inadequate living conditions in many cities, some people are unable to abide by physical distancing measures mandated by the federal and provincial governments [75]. Many people do not have access to financial resources for social buffering (i.e., reducing negative experiences related to the pandemic), have inadequate access to hygiene facilities and housing, and face overcrowded shelter systems. Thus, people who are already experiencing health and social inequities bear a disproportionate burden of risk during the COVID-19 pandemic [76–78]. Recent Canadian media related to COVID-19 has exposed intersecting forms of inequities and stigma that have become both revealed and exacerbated. These stories feature advocates such as organizations (e.g., Vancouver Area Network of Drug Users; VANDU^2^), academic scholars, and health care workers, including nurses, who are speaking up about food insecurity, social isolation, drug overdose, and gender-based violence. For example, an article published in one of Canada’s national newspapers, *The Globe and Mail*, discusses how the “incidence of infection and mortality from COVID-19 have shone a bright light upon health inequity in Canada” and specifically references how “the social, economic and environmental conditions of many Indigenous communities in Canada place First Nations, Inuit and Métis peoples at high risk for contracting COVID-19” [79].

These socio-political realities are at the heart of media items describing the profound impact of the COVID-19 pandemic on residents of an inner-city neighbourhood, the Downtown Eastside (DTES). This is a community recognized in Canada and across the globe as one of the most extreme examples of where health and social inequities co-exist alongside gentrification, wealth, and privilege. In the case of the DTES, media reports can serve to bring attention to existing inequities and injustice. For instance, through the media, we learned that gender-based and domestic violence within the community has increased during the COVID-19 era [80]. We also became aware that public health requirements to adhere to social distancing recommendations have had serious implications for women in this community, who are often faced with the dilemma of living with a partner who may perpetrate violence or increasing their risk of contracting the virus if they flee from their home [81].

At the same time, however, media representation can also lead to the perpetuation of stigma through decontextualized reporting. Recognizing that the role of media is to seek out and feature compelling stories (and these are often the dramatic, tragic, and disturbing), questions remain about the potential harms of decontextualized media reports that portray the negative effects of health and social inequities as taken-for-granted. In the case of a recent media item concerning a deceased newborn baby who was found in one of six temporary washrooms erected as part of the pandemic response in the DTES [82], we are concerned about the lack of attention to the range of services and resources that are urgently needed to support the health of women experiencing the intersecting issues of trauma, violence, and poverty – beyond the provision of port-a-potties. Viewing this tragic event through an equity lens, while being informed by structural violence, raises several questions. What actions are needed to address the impacts of gender-based violence and unsafe housing conditions for women in the DTES and other neighbourhoods that have high poverty rates? How could this media item be framed in ways that pre-empt proliferation of decontextualized analyses? And more specifically, what role might nurses play in influencing the ways in which media portray the impacts of health and social inequities during a pandemic?

Deepening an understanding of how inequities are framed, and how stigma is perpetuated through media, is relevant and critically important for nurses, and to improve health services generally. This understanding can feed into nurses’ crucial role in harnessing their energy to shape public discourses about inequities in our society. Amplifying the voices of direct care providers as expert sources in media - specifically the voices that counteract negative stigmatizing discourses - is an avenue toward improving health outcomes and care. By counteracting stigmatizing discourses, nurses can validate patient experiences and illuminate both the structural causes of ED pressures as well as the resulting impacts on people experiencing inequities. We recommend that nurses gain, and be provided with the necessary training to gain, the critical media skills needed to both review and contribute to media. For example, some non-profit organizations offer media training and media engagement resources to amplify the voices of experience-informed and diverse women in Canadian news media [83]. Publicly shared opinion pieces do not necessitate authorship from a person in a position of power, rather this form of communication can also represent the voices of those who provide direct care and bear witness to the many inequities that transpire on a daily basis. Drawing greater attention to these inequities could enhance public support for changes in systems affecting ED care and could influence policy makers toward making equitable care in EDs a higher priority.

There are also implications of our findings for people working outside of direct care. Upper-level health care management and regional health authority leadership should support nurses and other health care providers to engage with media through the provision of comprehensive media relations guidelines and related training. They should also identify a pool of nurses who are willing, and are trained to effectively speak from their positions. News journalists must be encouraged to prioritize building relationships with the nursing profession to include the voices of nurses in news and media.

### Limitations

While the goals of our study provided an opportunity to review media associated with EDs that brought forth issues related to social justice, equity, and inequity, a limitation of our analysis is related to the study context; specifically, our analysis was centered on three ED settings. The functioning of these three EDs, and how the media portrays ED care to the public, is deeply structured by the Canadian health care system and current socio-political structures. Though our study identified implications that may have relevance for other contexts, jurisdictions, or parts of the world, and these are aligned with themes found by health care media content analysis studies elsewhere [47], further research is needed, particularly in the era of (and following) the COVID-19 pandemic. In relation to criteria for rigour in qualitative work, the notion of transferability is relevant, that is, the extent to which the results of a qualitative study are applicable across different contexts, populations, or settings [84, 85]. Our work contains insights that readers may apply to their own local context. Future studies could expand the context of inquiry to include media portrayals of an increased number of EDs, and of health care settings beyond EDs, such as other hospital departments and primary care settings.

## Conclusions

The key recommendations of our study are as follows:

- We recommend that nurses and other health care providers be provided with critical media skill training and guidelines to both review and contribute to media, so that they can help educate media and policy actors about how to talk about care recipients, providers, and care provision in a more equity-oriented way.
- The voices and perspectives of nurses should be prioritized as those of experts in public communications related to emergency department care provision, system pressures, and reform.
- Partnerships between academic researchers and clinical practitioners should be strengthened to increase nursing’s visibility and contributions to media.
- We recommend that there should be no mandating that nurses refrain from contributing to public messaging, nor should there be abridgement of freedom of expression. Rather, for nurses who wish to relay honest accounts of their experiences, there should be guidance about how to do so.

## Data Availability

The datasets used and/or analysed during the current study are available from the corresponding author on reasonable request.

## Abbreviations

BC: British Columbia
COVID-19: Corona Virus Disease 2019
DTES: Downtown Eastside
ED: Emergency Department
OD: Overdose
SMH: Surrey Memorial Hospital
SPH: St. Paul’s Hospital
UHNBC: University Hospital of Northern British Columbia

## Footnotes

1 For a person to experience disadvantage means that structural factors, rather than personal, are determining of inequitable experiences.

2 VANDU: “VANDU is committed to increasing the capacity of people who use illicit drugs to live healthy and productive lives. We do this by affirming and strengthening people who use illicit drugs to reduce harms both to themselves and their communities” [86].
